# New Insights into FAK Phosphorylation Based on a FAT Domain-Defective Mutation

**DOI:** 10.1371/journal.pone.0107134

**Published:** 2014-09-16

**Authors:** Xuqian Fang, Xiangfan Liu, Ling Yao, Changqiang Chen, Jiafei Lin, Peihua Ni, Xinmin Zheng, Qishi Fan

**Affiliations:** 1 Department of Clinical Laboratory, Ruijin North Hospital, Ruijin Hospital, Shanghai JiaoTong University School of Medicine, Shanghai, P. R. China; 2 Faculty of Medical Laboratory Science, Shanghai JiaoTong University School of Medicine, Shanghai, P. R. China; 3 Department of Biochemistry and Molecular Biology, Shanghai JiaoTong University School of Medicine, Shanghai, P. R. China; 4 Department of Molecular Biology and Genetics, Cornell University, Ithaca, New York, United States of America; University of Iowa, United States of America

## Abstract

Mounting evidence suggests that the FAK N-terminal (FERM) domain controls FAK phosphorylation and function; however, little is known regarding the role of the C terminal (FAT) domain in FAK regulation. We identified a patient-derived FAK mutant, in which a 27-amino acid segment was deleted from the C-terminal FAT domain (named FAK-Del33). When FAK-Del33 was overexpressed in specific tumor cell lines, Y397 phosphorylation increased compared with that observed in cells expressing FAK-WT. Here, we attempt to unveil the mechanism of this increased phosphorylation. Using cell biology experiments, we show that FAK-Del33 is incapable of co-localizing with paxillin, and has constitutively high Y397 phosphorylation. With a kinase-dead mutation, it showed phosphorylation of FAK-Del33 has enhanced through auto-phosphorylation. It was also demonstrated that phosphorylation of FAK-Del33 is not Src dependent or enhanced intermolecular interactions, and that the hyperphosphorylation can be lowered using increasing amounts of transfected FERM domain. This result suggests that Del33 mutation disrupting of FAT's structural integrity and paxillin binding capacity leads to incapable of targeting Focal adhesions, but has gained the capacity for auto-phosphorylation in cis.

## Introduction

Focal adhesion kinase (FAK) plays a key role in promoting adhesion, proliferation, and cell migration and in regulating anchorage-dependent anti-apoptotic signals [1]. In addition, FAK is associated with tissue functions, such as vascular development, axon outgrowth, and dendrite formation, and diseases, such as cardiomyocyte-induced hypertrophy, fibrosis, and epithelial cancer [2], [3].

Auto-phosphorylation at Y397 is a key event in FAK activation [4]. Y397 phosphorylation creates a high-affinity binding site for the SH2 domain of Src family kinase, which leads to Src recruitment and activation [5]. Src recruitment facilitates FAK phosphorylation at tyrosine residues 576/7, 861, and 925, resulting in the activation of other focal adhesion proteins, such as paxillin [6]. Y397 auto-phosphorylation allows FAK to function as an adapter protein that communicates with various signal transduction pathways.

Selective FAK activation depends on the structure of FAK. FAK contains an N-terminal FERM domain, a central tyrosine kinase domain (KD), a C-terminal focal adhesion targeting (FAT) domain, a linker region between the FERM domain and the KD, and an unstructured proline-rich (PR) region between the KD and the FAT domain [7]. The current FAK signaling model suggests that FAK is inactivated by autoinhibition of the FERM domain and is activated by directly binding partner proteins and/or phosphoinositides located in a precise spatial and temporal manner [8]. Several FAK-interacting proteins, such as β-integrin, ezrin [9], EGFR [10], and C-Met [11], are responsible for FAK activation through FERM autoinhibitory conformational changes. The biological functions of these proteins widely vary, and in most cases, the FAK-interacting proteins that regulate biological activities remain poorly understood.

The C-terminal FAT domain has multiple protein-protein interactions sites (residues 919-1039 in human and murine FAK) that are critical for the recruitment of FAK to focal adhesion complexes, and Tyr925 is located within this domain [12]. FAK localization to focal adhesions is essential for integrin-dependent FAK regulation and FAK-mediated tyrosine phosphorylation of downstream substrates [13], [14]. Structures of the isolated FAT domain have demonstrated that the FAT domain forms an anti-parallel four-helix bundle that exists as a monomer and as a dimer due to domain swapping in helix 1 [15], [16]. The biological significance of this observation is not clear. Prutzman et al. [4] identified a FAT domain mutant (V955A/L962A) that dimerizes ∼8-fold more effectively than the WT-FAT domain and exhibits increased Tyr925 phosphorylation both *in vitro* and *in vivo*. These researchers suggest that the structural dynamics of the FAT domain may play an important role in determining FAK function.

In a previous study, we identified the novel splicing mutation FAK-Del33 in both breast and thyroid cancers through colony sequencing. The FAK-Del33 mutation frequency is 14.2% (3/21) in breast carcinomas [17]. This mutation completely deletes exon 33, causing loss of a small peptide (residues 969-995 in human FAK). Exon 33 is located in the C-terminus of FAK, which forms a FAT domain consisting of exons 32 and 34. Corsi [18] has reported various alternative exons, such as 13, 14, 16, and 31. Exons 13 and 14 are located near the kinase domain, whereas exon 31 is a short peptide located upstream of the FAT domain. The presence of a mutation in this region has not been identified in human tissues.

Several alternative exons coding for short peptides have been reported in rat and mouse FAK [19], [20]. However, most of these transcripts appear to be expressed at low levels in *Homo sapiens*, which suggest that these transcripts may play a regulatory role in transcription. Unlike these alternative variants, FAK-Del33 displays a relatively high abundance in tumor tissue, with 30-50% of samples containing mutant transcripts. These results indicate that FAK-Del33 may be related to cancer progression.

FAK tyrosine phosphorylation plays an important role in regulating FAK-mediated signaling. In the present study, we report that FAK phosphorylation at Y397 was increased in several tumor cells. FAK activation can be achieved through Src-dependent integrin or growth factor receptor stimulation or through trans-phosphorylation by other kinase [21], [22]. We discuss these two possibilities and suggest that FAK-Del33 is auto-phosphorylated. However, we propose that FAK-Del33 activation is not achieved by integrin stimulation or Src-dependent auto-phosphorylation. After assessing the effects of the FERM domain on FAK-Del33 phosphorylation, we hypothesize that the FAK-Del33 mutation potentially gained the capacity for autophosphorylation in cis.

## Materials and Methods

### Cell lines

MDA-MB-435s, MDA-MB-468, MDA-MB-231, HepG2, Hep3B, NIH3T3, Cos7, and REF cell lines were purchased from ATCC (Manassas, VA, USA) and cultured as recommended.

### Colonies sequencing of FAK

For the mutation analysis, fresh tissues (paired tumor/normal tissues) from breast cancer patients were collected after surgery and subjected to RNA extraction using the Trizol reagent (Invitrogen; CA, USA). RT-PCR was used to amplify the coding region of oligo-dT primed FAK cDNAs from the tissue specimens, and the products were cloned into pCR2.1 vectors. Ten positive colonies were selected from each sample and sequenced.

### Structural and protein alignment methods

To illustration of the 3D structure of the FAT domain, the software of pymol was used. For the FAK-WT and FAK-Del33 amino acid residue alignments, the online software of BLAST (bl2seq) was used. The reference FAK cDNA sequence was downloaded from CCDS Sequence Data for PTK2 [Homo sapiens (human)] with Gene ID: 5747 in NCBI website.

### Expression plasmid construction

The pCR2.1-FAK-Del33 plasmid, which contains the human FAK splicing mutation sequence fused to an HA tag, has been previously described [17]. DNA containing the entire FAK-Del33 coding sequence flanked by HindIII sites was generated from the pCR2.1-FAK-Del33 plasmid by PCR using Taq polymerase, and the cloning primers listed in Table S1. This DNA was cleaved with HindIII and ligated into pTet-Splice (Clontech, for Tet-off inducible expression) to create the pTet-FAK-Del33 plasmid, which allows for the inducible expression of HA-tagged FAK-Del33 or wild-type FAK.

### Generation and growth of inducible FAK-overexpressing cells

Cells were co-transfected with pSV2neo, pTet-FAK, or pTet-FAK-Del33, or with the empty pTet-Splice vector as a negative control. G418-resistant colonies were cloned and tested for inducible FAK expression. The cells were maintained in Dulbecco's modified Eagle medium (DMEM) supplemented with 300 mg/ml penicillin, 100 mg/ml streptomycin, 10% calf serum, and 5 ng/ml Dox (to suppress FAK expression). FAK expression was induced by growth without Dox for 16–18 h prior to the experiments.

### Cloning and mutagenesis for transient transfection

The PCDNA3.1^+^-GFP plasmid was a generous gift from Dr. Peihua Ni (University of Shanghai Jiaotong). DNA containing the entire FAK-Del33 coding sequence flanked by KpnI and NotI sites was generated from the pCR2.1-FAK-Del33 plasmid by PCR using Taq polymerase and the cloning primers are listed in Table S1. This DNA was cleaved with KpnI and NotI and then ligated into the pCDNA3.1^+^-GFP vector to generate GFP-FAK-Del33 plasmids, which expresses GFP fused to the C-terminal of FAK-Del33.

Using the same strategy, pCR2.1-FAK-Del33 was cleaved with NotI and NheI and then sub-cloned into PCDNA3.1^+^, and this plasmid resulted in overexpression when transiently transfected.

FAK-Del33 containing the Y397F mutation was generated by two-step PCR as follows (Fig. S1A). One PCR fragment was generated by amplifying pCR2.1-FAK-Del33 with the forward mutagenesis primer FAK Primer 1 (5′-TCA GAA ACA GAT GAT TTT GCT GAG ATT ATA GAT GA-3′, Flag-tag in the 5′ primer) and FAK NotI (5′-ATA AGC GGC CGC TCA AAT CTC AGC ATA ATC ATC TGT-3′). The other PCR fragment was generated by amplifying pCR2.1-FAK-WT/pCR2.1-FAK-Del33 with FAK 5′ BamHI (5′-GTC GGA TCC ATG GAT TAC AAG GAT GAC GAC GAT AAG GCA GCT GCT TAC CTT GAC-3′, Flag-tag in the 5′ primer) and the corresponding reverse mutagenesis primer (FAK Primer 2 5′-AGC AAA ATC ATC TGT TTC TGA CAC AGA GA-3′). The two purified fragments were mixed and amplified using the FAK 5′ BamHI and FAK 3′ NotI primers, and the resulting fragment was cloned into pCDNA3.1^+^.

A similar mutagenesis strategy was used for K454R (Fig. S1B). The forward mutagenesis primer was FAK Primer 3 (5′-CGG TTG CAA TTA GGA CAT GTA AAA ACT GT-3′), and the corresponding reverse mutagenesis primer was FAK Primer 4 (5′-ACA TGT CCT AAT TGC AAC CGC CAA AGC-3′). The FAK/Y397F or FAK-Del33/Y397F plasmid was used as a template for the K454R mutation. The resulting fragment was cloned into PCDNA3.1^+^.

The expression of FAK cDNA for transient transfection was created by amplifying pCR2.1-FAK-WT or pCR2.1-FAK-Del33 using the following primers: FAK 5′ BamHI (5′-GTC GGA TCC ATG TAC CCA TAC GAC GTG CCA GAC TAC-3′, HA tagged in the 5′ primer) and FAK 3′ NotI. The resulting PCR fragment was cloned into pCDNA3.1^+^.

The FAK deletion mutants Δ375-FAK and Δ375-FAK-Del33 were created by amplifying pCR2.1-FAK-WT or pCR2.1-FAK-Del33, respectively, using the Δ375F (5′-GTC GGA TCC ATG TTG GCC AAC AGC GAA AAG CAA GGC ATG-3′) and FAK NotI primers. The resulting PCR fragment was cloned into pCDNA3.1^+^.

The 1–396 FAK (FERM) fragment was created by amplifying pCR2.1-FAK-Del33 using the FAK 5′ BamHI primer (5′-GTC GGA TCC ATG GAT TAC AAG GAT GAC GAC GAT AAG GCA GCT GCT TAC CTT GAC-3′, Flag tag in the 5′ primer) and the FAK NotI primer. The resulting PCR fragment was cloned into pCDNA3.1^+^.

### Antibody and immunoblotting

Proteins were extracted from cells using lysis buffer for Westernblotting (Beyotime, Shanghai, China) supplemented with 1% protease inhibitor cocktail (Sigma-Aldrich, MO, USA), 25 mM NaF, and 1 mM Na_3_VO_4_. The proteins were separated using 8% mini SDS-PAGE for 1 h and transferred to PVDF membranes. The membranes were blocked in 5% BSA and Tris-buffered saline supplemented with 0.05% Tween-20 for 2 h at room temperature and then incubated overnight with specific antibodies, including anti-Y397, anti-FAK, and anti-GAPDH antibodies (CST, BL, USA). Anti-HA and anti-FLAG epitope (DYKDDDDK) monoclonal antibodies were purchased from Sigma-Aldrich.

### Biochemical treatment

For experiments where Cells replated on FN, ColI or poly-L-lysine were added to plates coated with 10 µg/ml FN, or 2 µg/ml Col I or 100 µg/ml poly-L-lysine for 60 min after being held in suspension. For experiments where cells were treated either the Src inhibitor PP2 or the control compound PP3, cells were plated onto FN-coated plates and transfected with indicated plasmids for 24 h. After that, Src inhibitor PP2 or the control compound PP3 were added to these cells for further 12 h before being lysed.

### Cell transfection and Immunofluorescence

Cells were transfected with 1 µg of DNA per well in 12-well plates, or with 2 µg of DNA per well in 6-well plates using Lipofectamine 2000 (Invitrogen, CA, USA) according to the manufacturer's instructions. Twenty-four hours after transfection, the cells were plated on glass coverslips and then fixed 24 h later for 20 min with PBS containing 4% (w/v) paraformaldehyde. After three rinses in PBS, the cells were permeabilized for 10 min with 0.2% Triton X-100 in PBS, treated with blocking buffer (PBS containing 5% w/v BSA) for 30 min and then incubated overnight with antibodies against paxillin or p-paxillin (Y118) at 4°C in PBS containing 1% BSA. After three rinses, the cells were sequentially incubated for 1 h at room temperature with goat Alexa-555-coupled anti-mouse antibodies. After three rinses, the cells were stained with DAPI (Invitrogen, CA, USA). Images were acquired at the Cell Imaging Facility using a laser scanning confocal microscope (Leica and SP5).

### Statistical analyses

The statistical significance between the data sets was determined using Student's t-test (SPSS software). The differences were considered significant when the *P*-value was less than 0.05.

## Results

### FAK-Del33 is hyper-phosphorylated in a cell line-dependent fashion

**Figure 1:**
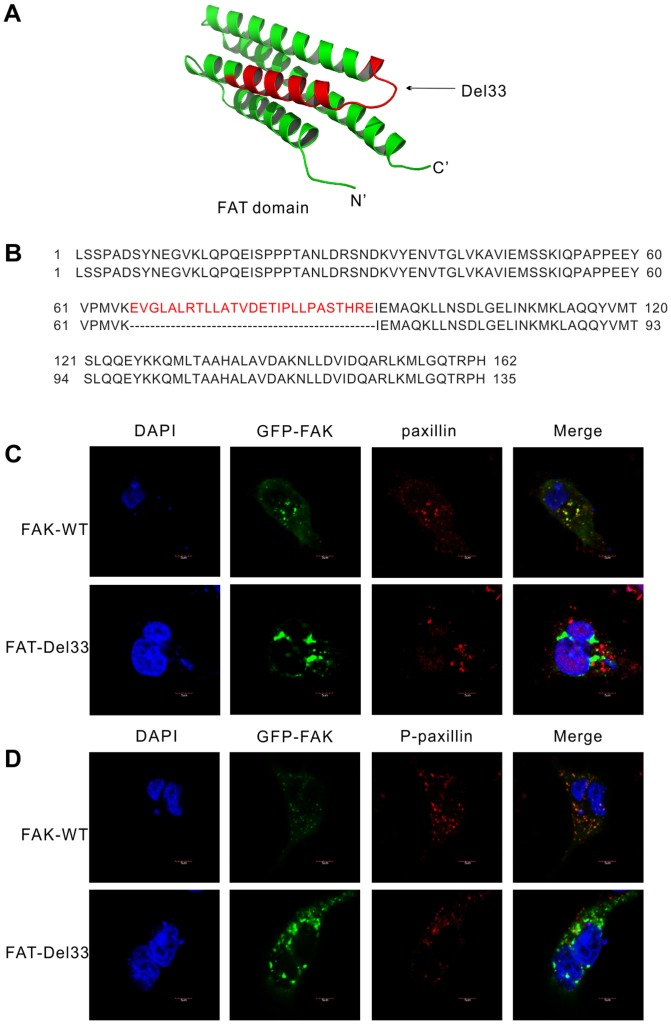
Structure and localization of FAK-Del33. (A) The 3D structure of the FAK FAT domain is shown as a ribbon representation (green) with the deleted portion of Del33 shown in Red. (B) FAT sequence conforms to Fig 1A was shown, with the deleted residues labeled in red. The protein sequence alignment shows that 27 amino acids (969-995Aa) are missing, but an ORF reading frame shift does not occur in FAK-Del33. (C, D) Confocal sections of MDA-MB_468 cells transfected with GFP-tagged FAK or GFP-tagged FAK-Del33. Cells were replated onto glass coverslips, and transfected for 24 h after which time they were fixed and processed for indirect immunofluorescence against either paxillin (C), or p-paxillin (D). Scale bar, 5 µm.

FAK-Del33 was isolated from breast and thyroid cancers harboring a complete deletion of exon 33, resulting in the absence of a small peptide (residues 969-995 in human FAK). An illustration of the 3D structure of the FAT _(901-1065)_ domain is shown in Fig 1A, where the deleted portion Del33 is highlighted in red. The FAT domain adopts an anti-parallel four-helix bundle, and the Del33 mutation happened in the second and third bundles. The amino acid alignment is shown in Fig 1B. Structural studies showed a minimal paxillin-binding region of FAK that encompasses residues 919-1042. The Del33_ (969-995)_ mutation is included in the minimal paxillin-binding region of FAK. Because FAT recruits FAK to adhesions by an interaction with paxillin and talin, we therefore expected that Del33 mutation would prevent the recruitment of this mutant to FAs. To test this hypothesis, we examined the cellular location of GFP-tagged FAK in epithelial tumor cells MDA-MB-468. In GFP-FAK-WT transfected cells, confocal analysis revealed that GFP fluorescence was co-localized with paxillin (Fig 1C, upper panel). Unlike the wild type protein, FAK-Del33 exhibited clustered, strong signals in the cytoplasm and failed to co-localize with paxillin (Fig 1C, lower panel). According to p-paxillin staining (Fig 1D), GFP-FAK was still co-localized with p-paxillin. However, cells expressing FAK-Del33 exhibit much lower p-paxillin signals, and these two proteins were not co-localized yet. It was demonstrated that paxillin was phosphorylated by FAK at Tyr118. Thus, we supposed Del33 mutation prevent the binding ability with paxillin, and further lost capacity to phosphorylated paxillin at Tyr118. This idea was also confirmed by CO-IP experiment (Data was not shown). So these results show that Del33 mutation completely abolish FAT's capacity to interact with paxillin.

**Figure 2:**
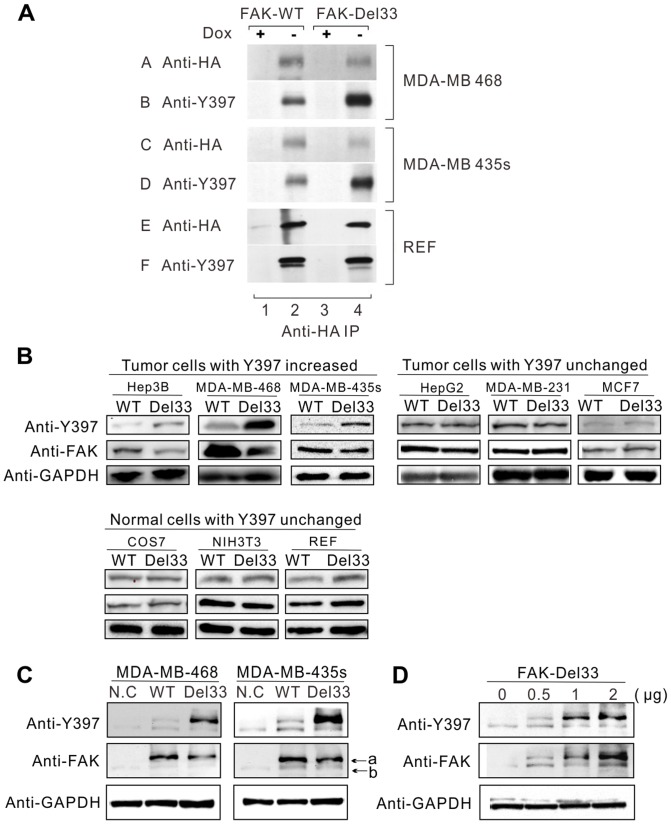
FAK-Del33 is hyper-phosphorylated in a cell-line dependent fashion. (A) Lysates containing 1 mg of total cellular protein were prepared from HA-tagged FAK-WT (lanes 1-2) and FAK-Del33 stable transfected cells (lanes 3-4). The cells overexpressing FAK were grown in the absence (induction of expression, even-numbered lanes) or presence (suppression of expression, odd-numbered lanes) of 5 ng/ml doxycycline (Dox). The lysates were immunoblotted with either anti-HA polyclonal (panels A, C, E) or anti-Tyr397 monoclonal (panels B, D, F) antibodies. (B) Different cells lines were transfected with each of the pCDNA3.1^+^-FAK-WT/FAK-Del33 plasmids separately. Equal amounts of the cellular lysates were subjected to Western blotting using the indicated antibodies. (C) The cells were transfected with pCDNA3.1^+^-GFP-FAK-WT/FAK-Del33 plasmids. A GFP tag was added to the N-terminus of FAK, and the resulting fusion protein has a larger mass than endogenous FAK (Arrow a shows GFP-FAK, arrow b shows endogenous FAK). (D) The cells were transfected with increasing amount of pCDNA3.1^+^-GFP-FAK-Del33 plasmids (0, 0.5, 1.0, 2.0 µg in 6-well plates.). The phosphorylation of endogenous FAK does not change with increasing expression and phosphorylation of exogenous FAK-Del33 in cells.

Cells expressing inducible HA-FAK-WT or HA-FAK-Del33 were generated as described in the Materials and Methods section. To investigate the potential role of the FAK-Del33 mutation in FAK tyrosine phosphorylation, the transfected cells were lysed, and the FAK proteins were immunoprecipitated using an HA antibody. Immunoblotting with an anti-HA antibody demonstrated that the expression of FAK increased to half-maximal levels 6–8 h after the removal of doxycycline (data not shown) and achieved maximum levels by 16–18 h (Fig 2A, panel A). Negligible protein levels were detected by the HA and Y397 antibodies in the presence of doxycycline (Fig. 2A, odd-numbered lanes). Analysis of immune complexes by Western blotting using an HA antibody (Fig 2A, panels A, C, E) indicated that exogenously expressed FAK and FAK-Del33 displayed similar expression levels. However, Y397 staining indicated that FAK-Del33 expression resulted in increased tyrosine phosphorylation in MDA-MB-435s and MDA-MB-468 cells. The Y397 levels were increased approximately 4.0- to 5.0-fold in FAK-Del33 overexpression cells compared with those expressing FAK-WT. Interestingly, this phenomenon did not occur in REF cells.

Given that the generation of inducible cell lines is time consuming and labor intensive, we expanded our analysis using the pCDNA3.1^+^ transient transfection system. We used the following cell lines: breast cancer cell lines MDA-MB-468, MDA-MB-435s, MDA-MB-231, and MCF7; liver cancer cell lines HepG2 and Hep3B; and normal cell lines REF, Cos7, and NIH3T3. The results shows that Y397 phosphorylation increased when FAK-Del33 was expressed in Hep3B, MDA-MB-468 and MDA-MB-435s cells, and similar tyrosine phosphorylation levels were maintained when FAK-Del33 was expressed in the other cell lines (Fig 2B). Interestingly, the FAK-Del33 mutation exclusively influenced tyrosine phosphorylation in cancer but not normal cell lines. We hypothesize that additional tyrosine kinase or activated signals in certain tumor cells may participate in FAK-Del33 mutation-induced Y397 phosphorylation.

To determine the influence of exogenously expressed FAK-Del33 on endogenous FAK phosphorylation, we constructed GFP-FAK, which can be readily distinguished from exogenous FAK by a gel shift assay. As presented in Fig 2C, FAK-Del33 expression in breast cells resulted in significant phosphorylation of exogenously expressed FAK-Del33, but not of endogenous FAK. To clarify this finding, increasing amounts of exogenous FAK were transfected in MDA-MB-468 cells to observe the phosphorylation status of endogenous FAK. In normal culture conditions, endogenous FAK is slightly phosphorylated. When FAK-Del33 was increasingly expressed and phosphorylated in cells, the phosphorylation status of endogenous FAK did not change at least in our experiment (Fig 2D). This result indicates that FAK-Del33 does not affect endogenous FAK phosphorylation. Thus, does endogenous FAK prefer phosphorylated FAK-Del33 compared with exogenous FAK-WT? We did not investigate this question because the FAK-knockdown cell line is not available in our lab.

Based on the current knowledge, FAK activation (measured by Y397 auto-phosphorylation) is achieved by two different mechanisms: 1) following integrin or growth factor receptor activation, Src binds auto-phosphorylated Y397, and the FAK-Src complex further phosphorylated additional Tyr residues within FAK [23], [24]; and 2) trans-phosphorylation by other kinase (e.g., overexpression of v-Src) results in the trans-phosphorylation of FAK Y397 [22], [25]. The FAK-Del33 mutation is found in the core sequence of the FAT domain, and this mutation may not localize to focal adhesions and is activated by an uncharacterized mechanism. We further assessed these two possibilities.

### FAK-Del33 is phosphorylated via enhanced auto-phosphorylation

**Figure 3:**
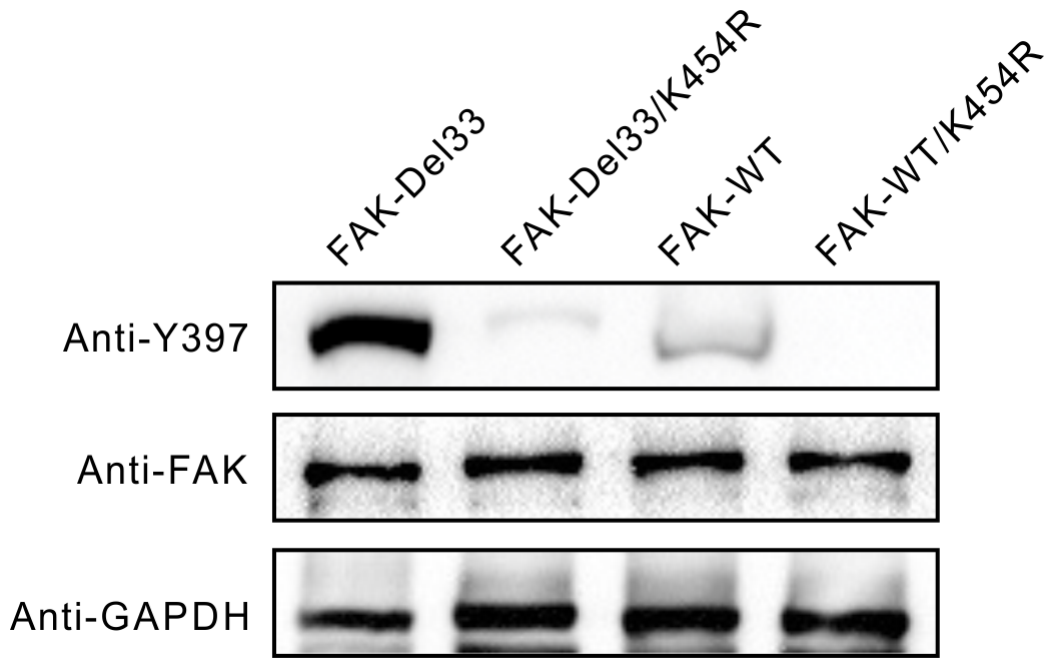
FAK-Del33 is phosphorylated due to enhanced auto-phosphorylation. MDA-MB-468 cells were transfected with 2 µg of plasmids encoding FAK-Del33, FAK-Del33/Y397F, FAK-WT, and FAK-WT/Y397F in 6-well plates. The total amount of DNA was kept constant by the addition of empty vector.

To determine whether FAK-Del33-induced phosphorylation resulted from auto- or trans-phosphorylation, we constructed the kinase-dead mutant K454R as described in the Materials and Methods section. Similar quantities of the FAK-Del33, FAK-Del33/K454R, FAK-WT, and FAK-WT/K454R plasmids were transfected in MDA-MB-468 cells. As expected, Y397 phosphorylation of FAK-WT/K454R dramatically decreased and was visible only after long exposure times (Fig 3, lane 2 and 4). Similar to FAK-WT, K454R in a FAK-Del33 background exhibited reduced Y397 phosphorylation levels (Fig 3, lane 1 and 3), suggesting that increased FAK-Del33 phosphorylation was dependent on its own activity, and that FAK-Del33 was not directly trans-phosphorylated by another tyrosine kinase. However, we should note that FAK-Del33/K454R phosphorylation remained increased compared with FAK-WT/K454R for unknown reasons.

### The FAK-Del33 mutation inhibits adhesion signal stimulation and induces constitutive phosphorylation

**Figure 4:**
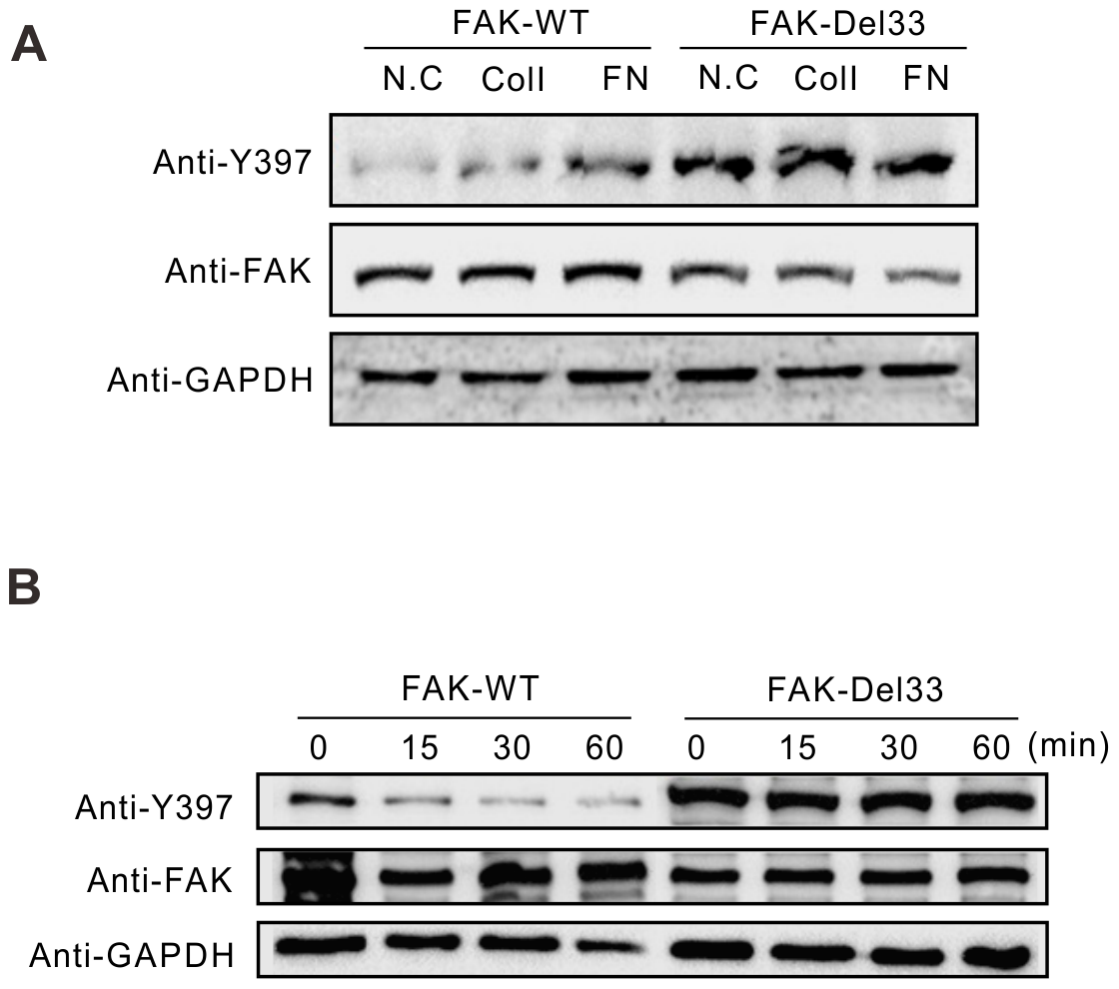
The FAK-Del33 mutation inhibits stimulation of adhesion signals and induces constitutive phosphorylation. (A) FAK-Del33 is insensitive to fibronectin or ColI treatment. MDA-MB-468 cells were transfected with FAK-WT or FAK-Del33 via viral infection and subjected to puromycin selection. The cells were trypsinized and suspended in DMEM with 0.1% BSA for 30 min before plating onto fibronectin (FN)- or ColI-coated dishes for 1 h. Cells plated on poly-L-lysine-coated dishes for 1 h were used as an untreated control (NC). (B) Time-dependent dephosphorylation of FAK-WT and FAK-Del33 in suspended cells. MDA-MB-468 cells stably transfected with FAK-WT or FAK-Del33 were trypsinized and suspended in serum-free medium for various times. Cell lysates were subjected to Western blotting using the indicated antibodies. FAK-Del33 phosphorylation persisted regardless of the suspension time course.

FAK is phosphorylated upon induction by integrin-mediated signaling, such as adherence to fibronectin- or ColI-coated plates, and this phosphorylation is abolished when cells are grown in suspension [26]. To determine whether FAK-Del33 is regulated by integrin-mediated signaling, we plated MDA-MB-468 cells stably expressing FAK-WT or FAK-Del33 (as described in [17]) on fibronectin (FN)- or ColI-coated plates for 1 h as indicated or on poly-L-lysine-coated dishes as the normal control (NC). As presented in Fig 4A, FAK-WT cells displayed increased phosphorylation levels after growth on ColI or FN, and the phosphorylation levels increased in the FN-treated samples. However, the phosphorylation in FAK-Del33-expressing cells was not altered upon cellular adhesion to FN. This indicates that the FAK-Del33 mutation inhibited adhesion signal stimulation.

In addition, cells were trypsinized and suspended in DMEM with 5% FBS for various time intervals. As expected, FAK-WT phosphorylation was reduced over time. However, FAK-Del33-overexpressing cells maintained increased levels of phosphotyrosine, even after incubation for 1 h at 37°C in suspension (Fig 4B). This result indicates that FAK-Del33 is constitutively phosphorylated independent of cell adhesion. Alternatively, FAK-Del33 dephosphorylation is delayed when cells are grown in suspension.

### The FAK-Del33 mutation reduces Src-mediated FAK phosphorylation

**Figure 5:**
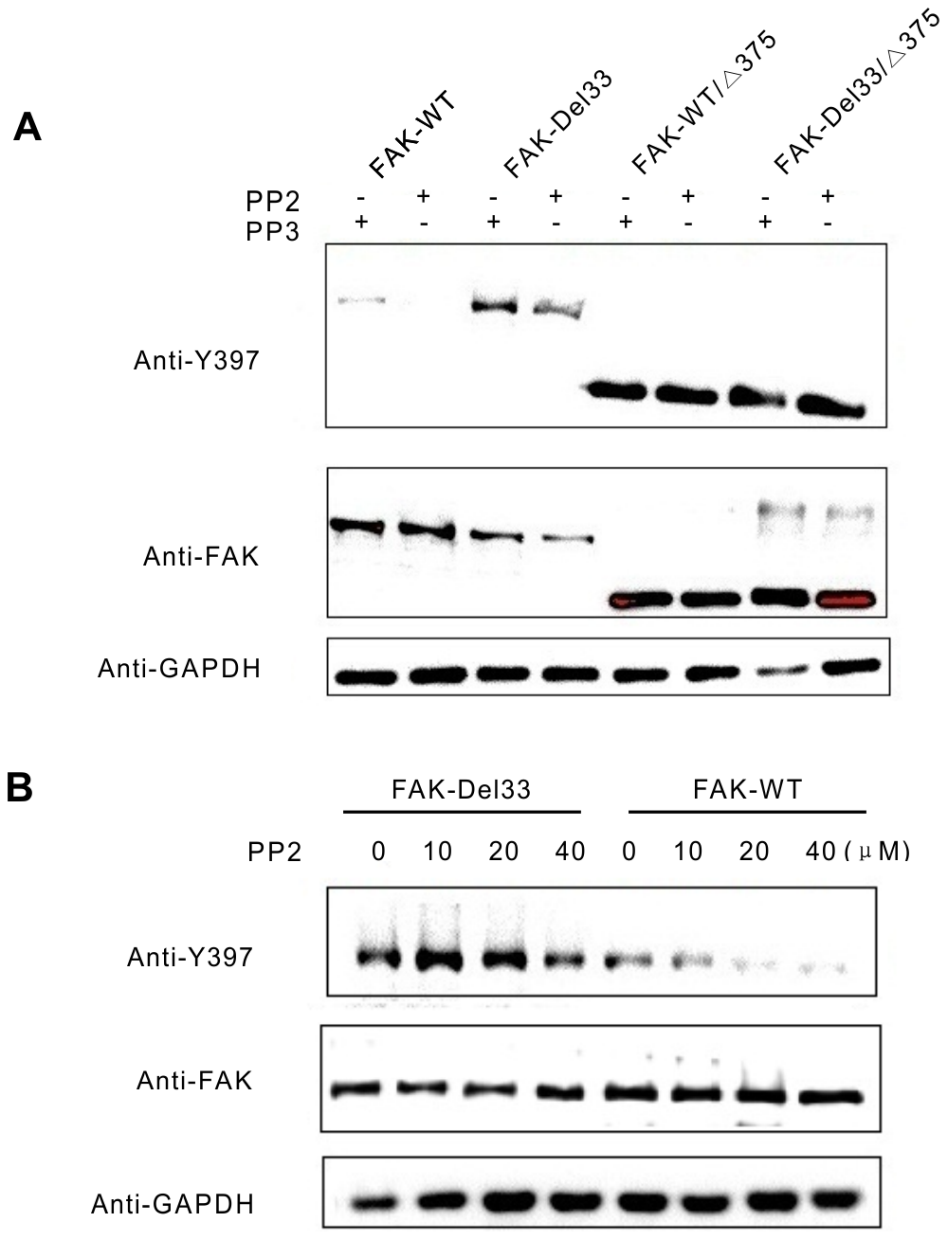
The FAK-Del33 mutation reduces Src independence. (**A**) MDA-MB-468 cells were transfected with FAK-WT, FAK-Del33, FAK-WT/Δ375, and FAK-Del33/Δ375. The cells were serum starved overnight and replated onto FN-coated plates in the presence of the Src inhibitor PP2 or the control compound PP3. (B) Cells transfected with FAK-WT or FAK-Del33 were treated with increasing concentration of Src inhibitor PP2 (0∼40 µm). Whole cell lysates were immunoblotted with antibodies against Y397 (top panel), FAK (middle panel), and GAPDH (bottom panel).

The tyrosine kinase Src is thought to play an integral role in regulating FAK phosphorylation. To clarify the role of Src in FAK-Del33 phosphorylation, we transiently transfected FAK-WT, FAK-Del33, FAK-WT/Δ375, and FAK-Del33/Δ375 truncation mutants into MDA-MB-468 cells. The cells were replated onto FN in the presence of the Src inhibitor PP2 (40 µM) or the control compound PP3 (40 µM). Similar to previous research [7], [27], Y397 phosphorylation was strongly reduced when cells expressing full-length, wild-type FAK were replated in the presence of the Src inhibitor PP2, but not when in the presence of the PP3 control (Fig 5A, lane 1 and 2). The FAK-WT/Δ375 and FAK-Del33/Δ375 constructs were used as negative control because it has been clearly demonstrated that the removal of the amino terminus through a truncation mutation renders FAK's dependency on the activity of Src (Fig 5A, lane 5-8). However, the effect of PP2 on the FAK-Del33 mutation was intermediate between its effects on full-length, wild type FAK and on the truncation mutant. Although PP2 reduced Y397 phosphorylation, the Del33 mutant remained phosphorylated (Fig 5A, lane 3 and 4) at increased levels compared with PP3-treated FAK-WT. Similar results were observed in the PP2 dose-response treatment (Fig 5B). FAK-WT or FAK-Del33 expressed cells were treated with increasing concentrations of the Src inhibitor PP2 (0, 10, 20, and 40 µM). As shown in the wild-type cells, PP2 treatment reduced FAK tyrosine phosphorylation in a dose-dependent manner, and the levels were clearly reduced in the 10 µM PP2 treatment. In contrast, FAK-Del33 phosphorylation was slightly altered when the cells were treated with 10 or 20 µM PP2, and the levels were reduced upon exposure to 40 µM PP2. In conclusion, the FAK-Del33 mutation weakened its Src-dependent activity.

### Intermolecular interactions do not play a dominant role in the auto-phosphorylation FAK-Del33

**Figure 6:**
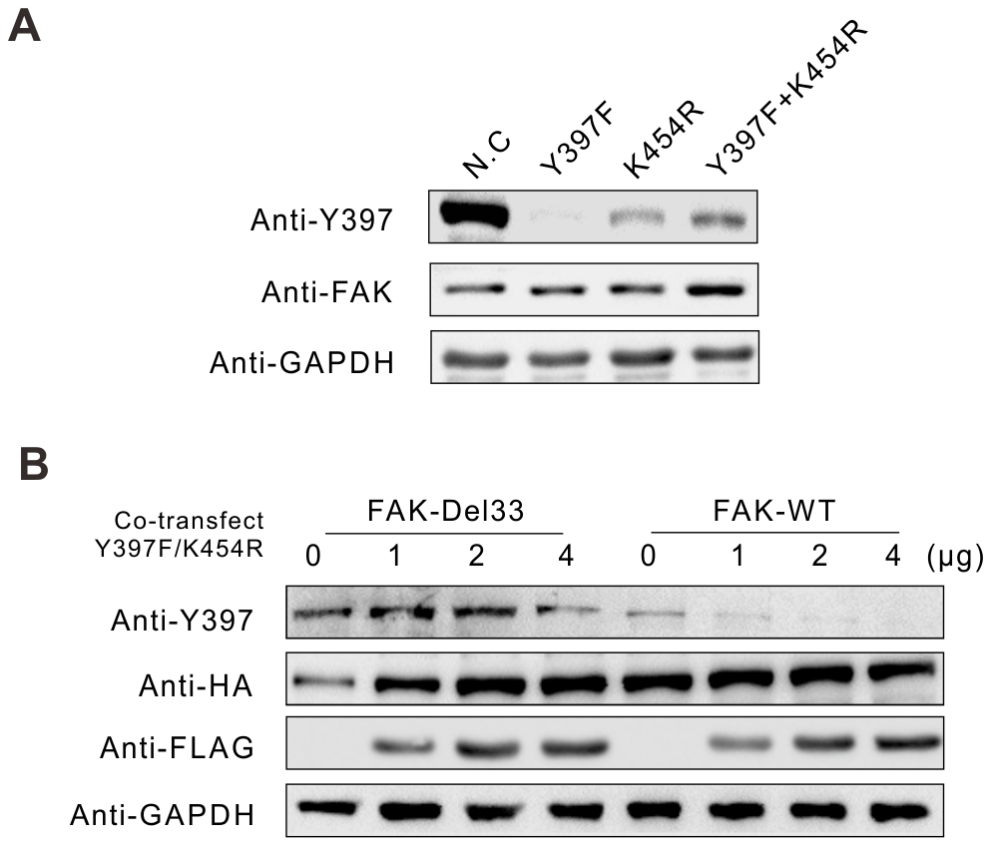
Intermolecular interactions do not play a dominant role in FAK-Del33 auto-phosphorylation. (A) MDA-MB-468 cells were transfected with 2 µg of plasmids encoding N.C, Y397F, K454R, or Y397F+ K454R with FAK-Del33 background in 6-well plates. The total amount of DNA was kept constant through the addition of empty vector. (B) MDA-MB-468 cells were transfected with FAK (2 µg) and increasing amounts of the FAK Y397F/K454R double mutant (0-4 µg, as indicated) in the FAK-Del33 and FAK-WT backgrounds. The total amount of DNA was kept constant through the addition of empty vector.

To determine the contribution of an intermolecular mechanism in the auto-phosphorylation of FAK-Del33, we constructed site point mutated (Y397F), kinase-dead (K454R), and a double mutant (Y397F/K454R) in the FAK-WT and FAK-Del33 backgrounds. The same quantity of N.C (plasmids containing FAK-Del33 without further mutation), Y397F, K454R, or both plasmids (Y397F+K454R) was transfected in MDA-MB-468 cells with the FAK-Del33 background (Fig 6A). Compared to the Y397 staining in the K454R lane, the lane containing Y397F+K454R shoes slightly increased phosphorylation. However, compared to the Y397 staining of the N.C lane, the lane containing Y397F+K454R only restored a small fraction of Y397 phosphorylation. This finding suggests that intermolecular phosphorylation plays a small role in FAK-Del33 auto-phosphorylation.

To determine whether auto-phosphorylation of FAK-Del33 is intermolecular, we also transfected MDA-MB-468 cells with FAK and increasing amounts of the Y397F/K454R double mutant. If FAK auto-phosphorylation occurs through an intermolecular reaction in intact cells, the expression of the Y397F/K454R double mutant will compete in the intermolecular auto-phosphorylation reaction. As expected, we observed a dose-dependent inhibition of Y397 phosphorylation on FAK-WT. However, Y397 phosphorylation of FAK-Del33 was minimally affected (Fig 6B). This result indicates that intermolecular interactions do not play a dominant role in the phosphorylation of FAK-Del33.

### Intramolecular interactions contribute to FAK-Del33 phosphorylation

**Figure 7:**
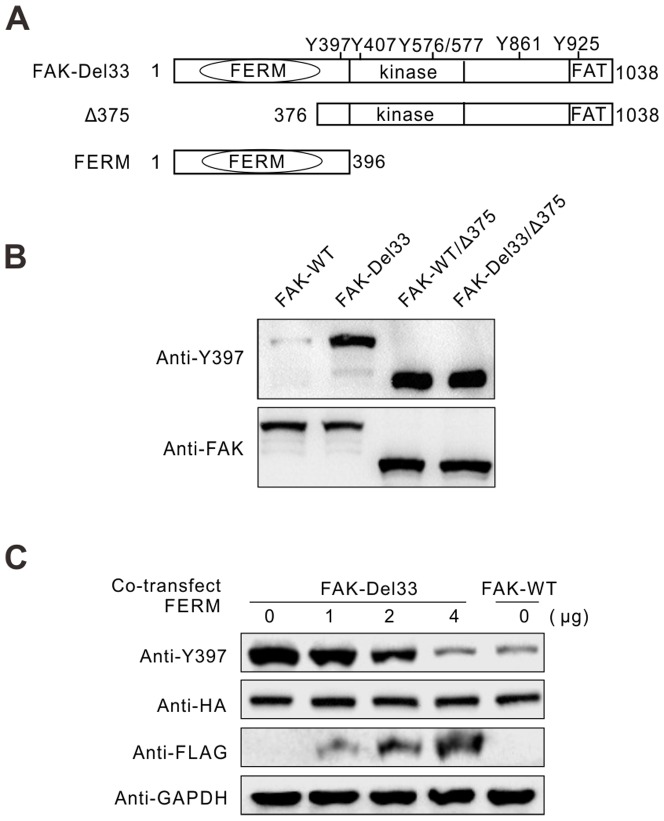
Intramolecular interactions contribute to FAK-Del33 auto-phosphorylation. (A) MDA-MB-468 cells were transfected with FAK or FAK/Δ375 plasmids. Then, 30 µg of whole cell lysates were immunoblotted with Y397 and total FAK antibodies. (B)The FERM domain inhibited Y397 phosphorylation in cis in FAK-Del33. MDA-MB-468 cells were co-transfected with HA-tagged FAK (2 µg) and increasing amounts of Flag-tagged FERM domain fragments (consisting of residues 1–396, 1-4 µg, as indicated). The total amount of DNA was kept constant by the addition of empty vector. In total, 30 µg of whole cell lysates was immunoblotted with the indicated antibodies.

To determine whether auto-phosphorylation of FAK-Del33 can be intramolecular, we depleted the FERM to remove FERM-Kinase intramolecular autoinhibition. We transiently transfected FAK-WT, FAK-Del33, FAK-WT/Δ375, and FAK-Del33/Δ375 truncation mutants into MDA-MB-468 cells. As expected, after FERM domain truncation, Y397 phosphorylation of FAK-WT/Δ375 increased sharply (Fig 7A, lanes 1 and 2). However, no apparent difference in Y397 phosphorylation was observed in FAK-Del33 expressing cells after truncation of the FERM domain (Fig 7A, lane 3 and 4), which indicates that the Del33 mutation disrupts the autoinhibitory conformation. Furthermore, the Y397 phosphorylation of FAK-Del33 was similar to that of FAK-WT/Δ375 if the exogenously expressed FAK was in similar expression levels, which indicates that FAK-Del33 phosphorylation was dominant contributed from intramolecular interaction.

How does the Del33 mutation disrupt FERM-mediated autoinhibition? One possible explanation is that the FERM domain lost its capacity to bind its kinase domain containing the Del33 mutation. To assess this possibility, we cotransfected MDA-MB-468 cells with HA-tagged FAK-Del33 and increasing amount of the FLAG-tagged amino-terminal FAK domain (residues 1 to 396; FERM). The cell lysates were subjected to immunoblotting with an anti-Y397 antibody to determine the cellular tyrosine phosphorylation levels. As shown in Fig 7B, FERM overexpression reduced tyrosine phosphorylation of the FAK-Del33 mutant in a dose-dependent manner; thus, FAK-Del33 is sensitive to FERM overexpression. When 4 µg of the FERM plasmid and 2 µg of FAK-Del33 were co-expressed in 6-well cell culture plates, Y397 phosphorylation decreased to the levels of the FAK-WT. This suggests that the default of FERM-kinase binding ability does not reasonable to explain hyperphosphorylation of this mutant.

## Discussion

In this study, we provide interesting insight into the possible mechanism of Y397 phosphorylation in a FAT domain-defective mutant (FAK-Del33). Similar to FAK-WT, Y397 auto-phosphorylation was reduced due to the catalytic domain inactive mutation. In contrast to FAK-WT, FAK-Del33 was constitutively phosphorylated and insensitive to adhesion signals, and Src-mediated regulation of FAK phosphorylation was clearly reduced. This result indicates that FAK-Del33 undergoes auto-phosphorylation, and this event is likely not achieved by integrin stimulation or Src-dependent auto-phosphorylation. Thus, other mechanisms may be involved.

A similar mutation with a deletion between residues 965-1012 (resulting from an artificial mutation) has been described by Shen Y [28], [29] and Hildebrand [29]. The mutation displays increased phosphorylation in culture similar to FAK-WT, and no increase upon fibronectin plating or difficulty in de-phosphorylation while in suspension is evident. In comparison, FAK-Del33 results in a deletion in residues 969-995 (isolated from tumor patient samples). This mutation confers increased phosphorylation (4-5 ratios) in certain cell lines compared with FAK-WT. The FAK-Del33 mutation also inhibits the stimulation of adhesion signals and induces constitutive phosphorylation; these activities are similar to the artificial 965-1012 deletion mutation. Until now, the mechanism by which the FAK 965-1012 deletion mutation becomes tyrosine phosphorylated has still been unclear. The author hypothesized that the mutant protein is unable to associate with the protein tyrosine phosphatase (PTP) responsible for FAK dephosphorylation; however, the author was unable to provide sufficient data to prove the hypothesis [28].

The mechanism of FAK auto-phosphorylation differs markedly from alternatively spliced isoforms [30]. FAK+6,7 (a major form of FAK in the brain) confers increased overall tyrosine phosphorylation due to the presence of additional residues on either side of Y397, thereby providing additional length and flexibility to the peptide chain and subsequently altering the autoinhibitory interactions [31]. The CD2-FAK fusion protein is permanently phosphorylated at tyrosine residues through constitutive recruitment of FAK to the plasma membrane [32]. Super-FAK, which contains an amino substitution for two lysine residues in the FAK activation loop, exhibits increased catalytic activity *in vitro* with enhanced biochemical signaling and cell motility [33]. K38A, which possesses a FERM mutation, displays increased phosphorylation independent of cell adhesion due to the disruption of autoinhibitory conformations [34]. Experiments using K454R demonstrate that Y397 phosphorylation in FAK-Del33 requires an intact kinase domain, thereby indicating that FAK-Del33 is auto-phosphorylated by its kinase domain rather than trans-phosphorylated by other kinase.

Two FAK auto-phosphorylation models currently prevail. One was proposed by Lietha [35], [36] that states that FAK phosphorylate itself intramolecularly. They found that the key initial step in FAK activation is the displacement of the FERM domain by competitive binding of an activating protein to the FERM F2 surface, which might then directly disrupt the FERM/kinase interface to allow for the rapid auto-phosphorylation of the linker residue Y397. In a subsequent step, the displacement of the FERM domain could represent an initial docking site for Src, which might then phosphorylated the activation loop residues Y576 and Y577 of FAK to yield full catalytic activity. The other model states that FAK is auto-phosphorylated mostly through an intermolecular mechanism. On the basis that FAK phosphorylation can be enhanced by induced dimerization [30], [37]. In further Brami-Cherrier [38] found that FAK dimerization plays dominant role in Y397 phosphorylation and that FAK dimers are stabilized by the FAT: FERM interaction.

For FAK-WT, we believe that the two FAK phosphorylation models may be mixed as Y397 phosphorylation can occur both in cis and in Trans. However, for FAK-Del33, which shows decreased intermolecular interactions (Fig 6), we proposed that intermolecular mechanism would not be the predominant element. A previous study by Toutant et al. [30] suggests that intermolecular interactions require the C-terminal region of FAK (841-1054). This region contains the FAK-Del33 deletion region 969-995. Our results support the notion that the FAK C-terminus plays important roles in intermolecular interactions. A potential explanation for these effects is that FAK-Del33 mutation leads to loss of the 3D fold of this domain, and hence of FAK functions requiring either the 3D fold, or the 33 amino acids that were deleted. For instance, Paxillin binding to FAT, which is a major driver of FAK localization at FAs, requires both the FAT 3D fold and the 33 amino acids deleted (they form the LD motif binding site located on helices 2/3) [30], [38]. According to Brami-Cherrier's study, FAT may participate in Y397 auto-phosphorylation by stabilizing the FAK dimers. A basic cluster on FERM that binds to FAT and stabilizes the FAK dimers has been revealed. Although the detailed amino sequence involved in FAT binding to FERM is still unclear, a minimal, 4-helix bundle (FAT 916–1024) FAT domain is sufficient for FERM binding. Furthermore, the binding of the LD motifs of paxillin did not decrease FAT binding to GST-FERM, but instead enhanced this binding. Therefore, it is very likely that the Del33 (969-995) mutation which destroys the binding to paxillin, would also destroy its stabilizing ability.

Work by Lietha et al. has shown that a FAK constructs which lacks the C-terminal region (and hence FAT) is capable of autophosphorylating in cis and trans [35]. Toutant et al. and Brami-Cherrier et al. have shown that full-length FAK (including the FAT domain) cannot autophosphorylate efficiently in cis, and needs dimerisation for autophosphorylation in trans [30], [38]. Dimerisation is enforced by interactions between the FAT domain of one protomer and the FERM domain of the other. In our experiment, intermolecular dimerisation does not play a dominant role in the auto-phosphorylation FAK-Del33. And also considering the dose dependent decrease caused by the FERM domain, we suppose FAK-Del33 mutant may act like the FERM-kinase fragment and have autophosphorylation in cis enabled. We expressed this mutant in different cell lines, and found that only some cell lines showed increased Y397 phosphorylation. We used to consider a FAK interacting protein involved in FAK-Del33 autophosphorylation, which may only be abundant on certain tumor cell lines. But we didn't get a candidate protein through pull down experiment. A potential explanation for this phenomenon is that the efficiency of this autophosphorylation in cis is modulated by cell-dependent factors, which may alter over-expression levels, local FAK-Del33 concentrations and/or FERM/kinase interactions.

In conclusion, our paper provides an explanation for why del33, which targets structure and function of the FAT domain, allows autophosphorylation in cis.

## Supporting Information

Table S1 **Primers used in this paper.**
(DOCX)
