# Supplementary material for: New Insights into FAK Phosphorylation Based on a FAT Domain-Defective Mutation
Source: PLoS One. 2014 Sep 16;9(9):e107134. doi: 10.1371/journal.pone.0107134 (PMC4166415; doi:10.1371/journal.pone.0107134)
Supplement: Table S1 — Primers used in this paper. (DOCX) [file pone.0107134.s001.docx]

**Supplement information**

**Table S1 Primers used in this paper**

| **No** | **Primers** | **Sequence** | **Purpose** |
| --- | --- | --- | --- |
| 1 | HA-FAK-F-KpnI | 5′-CGG GGT ACC ATG TAC CCA TAC GAC GTG CCA GAC TAC-3′ | GFP-FAK |
|  | HA-FAK-R-NotI | 5′-ATA AGC GGC CGC TCA GTG TGG TCT CGT CTG CCC AAG-3′ |  |
| 2 | Y397F-F | 5′-TCA GAA ACA GAT GAT TTT GCT GAG ATT ATA GAT GA-3′ | Y397F point mutation |
|  | Y397F-R | 5′-AGC AAA ATC ATC TGT TTC TGA CAC AGA GA-3′ |  |
| 3 | K454R-F | 5′-CGG TTG CAA TTA GGA CAT GTA AAA ACT GT-3′ | K454R point mutation |
|  | K454R-R | 5′-ACA TGT CCT AAT TGC AAC CGC CAA AGC-3′ |  |
| 4 | Δ375F | 5′-GTC GGA TCC ATG TTG GCC AAC AGC GAA AAG CAA GGC ATG-3′ | FAK/Δ375 |
|  | FLAG-FAK-R-NotI | 5′-ATA AGC GGC CGC TCA GTG TGG TCT CGT CTG CCC AAG-3′ |  |
| 5 | FLAG-FAK-F-BamHI | 5′-GTC GGA TCC ATG GAT TAC AAG GAT GAC GAC GAT AAG GCA GCT GCT TAC CTT GAC-3′ | FLAG tagged FERM |
|  | FLAG-FAK400-R-NotI | 5′-ATA AGC GGC CGC TCA AAT CTC AGC ATA ATC ATC TGT-3′ |  |
